# Measuring availability of and facility readiness to deliver comprehensive abortion care: experiences and lessons learnt from integrating abortion into WHO’s health facility assessments

**DOI:** 10.1136/bmjgh-2024-015097

**Published:** 2024-08-09

**Authors:** Heidi Bart Johnston, Katy Footman, Mohamed Mahmoud Ali, Eman Abdelkreem Aly, Chilanga Asmani, Sofonias Getachew Asrat, Dominic Kwabena Atweam, Sayema Awais, Richard Mangwi Ayiasi, Martin Owusu Boamah, Ovost Chooye, Roseline Doe, Benson Droti, Hayfa Elamin, Chris Fofie, Karima Gholbzouri, Azmach Hadush, Nilmini Hemachandra, Yelmali Hien, Francis Chisaka Kasolo, Hillary Kipruto, Yolanda Barbera Lainez, Nasan Natseri, Pamela Amaka Onyiah, Christopher Garimoi Orach, Assane Ouangare, Leopold Ouedraogo, Olive Sentumbwe-Mugisa, Ashley Sheffel, Amani Siyam, Martin Ssendyona, Ellen Thom, Rose Koirine Tingueri, Soumaïla Traoré, Qudsia Uzma, Wendy Venter, Bela Ganatra

**Affiliations:** 1 UNDP-UNFPA-UNICEF-WHO-World Bank Special Programme of Research, Development and Research Training in Human Reproduction (HRP), Department of Sexual and Reproductive Health and Research, World Health Organization, Geneva, Switzerland; 2 World Health Organisation Regional Office for the Eastern Mediterranean, Cairo, Egypt; 3 formerly, World Health Organization Regional Office for Africa, Brazzaville, Congo; 4 Ghana, World Health Organization, Accra, Ghana; 5 Pakistan Ministry of National Health Services Regulations and Coordination, Islamabad, Pakistan; 6 Public Health, Muni University, Faculty of Health Sciences, Arua, Uganda; 7 Zambia Ministry of Health, Lusaka, Zambia; 8 World Health Organization Regional Office for Africa, Brazzaville, Congo; 9 Family Health Division, Ghana Health Service, Accra, Ghana; 10 Zambia, World Health Organization, Lusaka, Zambia; 11 Myanmar, World Health Organization, Yangon, Myanmar; 12 Burkina Faso, World Health Organization, Ouagadougou, Burkina Faso; 13 International Health Consultant, Panama City, Panama; 14 Uganda, World Health Organization, Kampala, Uganda; 15 Makerere University College of Health Sciences, Kampala, Uganda; 16 Directorate of Statistics, Ministry of Health Burkina Faso, Ouagadougou, Burkina Faso; 17 Department of International Health, Johns Hopkins Bloomberg School of Public Health, Baltimore, Maryland, USA; 18 World Health Organization Regional Office for South-East Asia, New Delhi, New Delhi, India; 19 Department for Standards Accreditation and Patient Protection, Republic of Uganda Ministry of Health, Kampala, Uganda; 20 Pakistan, World Health Organization, Islamabad, Pakistan; 21 Data and Analytics, World Health Organization, Geneva, Switzerland

**Keywords:** Global Health, Health systems, Health systems evaluation, Maternal health, Public Health

## Abstract

Routine assessment of health facility capacity to provide abortion and post-abortion care can inform policy and programmes to expand access and improve quality. Since 2018, abortion and/or post-abortion care have been integrated into two WHO health facility assessment tools: the Service Availability and Readiness Assessment and the Harmonised Health Facility Assessment. We discuss lessons learnt through experiences integrating abortion into these standardised tools. Our experiences highlight the feasibility of including abortion in health facility assessments across a range of legal contexts. Factors facilitating the integration of abortion include cross-country collaboration and experience sharing, timely inputs into tool adaptations, clear leadership, close relationships among key stakeholders as in assessment coordination groups, use of locally appropriate terminology to refer to abortion and reference to national policies and guidelines. To facilitate high-quality data collection, we identify considerations around question sequencing in tool design, appropriate terminology and the need to balance the normalisation of abortion with adequate sensitisation and education of data collectors. To facilitate appropriate and consistent analysis, future work must ensure adequate disaggregation of recommended and non-recommended abortion methods, alignment with national guidelines and development of a standardised approach for measuring abortion service readiness. Measurement of abortion service availability and readiness should be a routine practice and a standardised component of health facility assessment tools. Evidence generated by health facility assessments that include abortion monitoring can guide efforts to expand access to timely and effective care and help normalise abortion as a core component of sexual and reproductive healthcare.

**Summary box**

- Abortion care has often been excluded from health facility assessments due to legal restrictions on abortion and stigma, resulting in a gap in information on service availability and facility readiness to provide what can be life-saving care.
- To inform future inclusion of abortion care services within health facility assessments, we reviewed experiences of including abortion indicators in health facility assessments in five countries and documented lessons learnt in areas of design, implementation and analysis.
- Our experiences highlight that it is feasible to include abortion in health facility assessments across a broad range of countries and legal contexts. Experience including abortion indicators in health facility assessments in multiple countries suggests several steps can be implemented to facilitate inclusion: use of locally appropriate terminology to refer to abortion, reference to policy affirming legal indications, appropriate sequencing of abortion questions in the assessment tool, enumerator training that balances normalisation of abortion with adequate sensitisation and strong working relationships among key stakeholders—including sexual and reproductive health experts—throughout the assessment process. Inclusion of abortion in health facility assessment core tools can help to normalise abortion as a routine and essential component of healthcare, help clarify the conditions under which the service can be provided and provide evidence for health system strengthening.

## Introduction

 Access to quality healthcare is central to universal health coverage. Health facility assessments externally review the facility capacity to deliver required health services and are an important component of health information systems. Some aspects of sexual and reproductive health are commonly included in health facility assessments,^1^ however, comprehensive abortion care, due to legal restrictions and stigma, is frequently excluded.^2^ Comprehensive abortion care includes the provision of information, abortion management (including induced abortion and care related to pregnancy loss) and post-abortion care (PAC). Post-abortion care includes the provision of services after an abortion, such as contraceptive services and linkage to other needed services in the community or beyond. It can also include the management of complications after an abortion.^3^ Where abortion and/or post-abortion care have been included in facility survey or census data, a signal functions approach^4^ has been used and analyses have highlighted low facility capacity in countries with abortion data.^5^,^16^ However, as abortion and post-abortion care are rarely included in health facility assessments, service coverage gaps can go undetected and unaddressed.^1^ Monitoring facility capacity for quality abortion care is crucial for policy and programme improvement, enhancing access and reducing morbidity and mortality from unsafe abortion complications.^17^ Although abortions (safe and unsafe) can happen outside health facilities, facilities remain vital for treating abortion complications, offering surgical methods and addressing additional medical, social and emotional needs.

### Integrating abortion into WHO health facility assessments

The WHO supports the use of comprehensive, standardised, health facility assessment tools—the Harmonised Health Facility Assessment (HHFA)^18 19^ and its predecessor, the Service Availability and Readiness Assessment (SARA)^20^; national Ministries of Health oversee the implementation of these tools. The SARA, initiated in 2008, shares most indicators with the HHFA, introduced in 2018. But the HHFA has expanded to cover management, finance and quality of care and uses a more automated approach for efficient data collection and analysis. Both tools generate tracer indicators to evaluate service availability and readiness, and are applicable in a representative sample or a census of facilities.

Since 2018, the WHO has been developing and testing an abortion section to include in the health facility assessments, with the aim to support Ministries of Health with evidence for decision-making, resource allocation and monitoring for comprehensive abortion care. The SARA historically has not included comprehensive abortion care, but abortion was included in SARA for the first time in Pakistan in 2020.^21^ Post-abortion care was included in the 2018/2019 HHFA in Kenya, and abortion or post-abortion care sections were subsequently included in HHFAs in Burkina Faso, Democratic Republic of the Congo, Ghana, Liberia, Maldives, Somalia, Uganda and Zambia. Abortion and post-abortion care were incorporated into the core HHFA tool in 2022. To inform future inclusion of abortion within health facility assessments, we documented lessons learnt from the process of design, implementation and analysis.

### Lessons learnt at each stage of development and implementation

To document lessons learnt we reviewed experiences in 5 of the 10 countries that have included abortion or post-abortion care in SARA or HHFA: Burkina Faso, Ghana, Pakistan, Uganda and Zambia (table 1). We selected these countries to document lessons from a range of abortion legal contexts, regions and time periods of implementation.

**Table 1:** Summary of selected countries in which lessons learnt were documented

Country	Pakistan	Burkina Faso	Zambia	Uganda	Ghana
Tool	SARA	HHFA	HHFA	HHFA	HHFA
Scope and timing	Pilot in January to February 2020 in Islamabad Capital Territory, implementation in 11 additional districts in November to December 2021	Census of health facilities in November 2020 to January 2021	Census of health facilities in November 2021 to January 2022	Nationally representative sample of facilities across all 15 subregions in April 2022	Nationally representative sample of facilities across all 16 regions in January to February 2023
Topics included in abortion section* †	Legally indicated induced abortion and post-abortion care	Legally indicated induced abortion and post-abortion care	Legally indicated induced abortion and post-abortion care	Post-abortion care only	Legally indicated induced abortion and post-abortion care
Legal status of induced abortion‡	Legal on the grounds of ‘necessary treatment’ or to protect the life of the woman^31 32^	Legal to protect the woman’s life or health and in cases of fetal impairment, rape or incest^33^	Legal to protect the woman’s life, physical or mental health and in cases of fetal impairment^27^	Legal to protect the woman’s life^34^	Legal to protect the woman’s life, physical or mental health and in cases of fetal impairment, rape or incest^35^
Institutional partners	Centre for Global Public Health-Pakistan, collaborative centre for Institute of Global Public Health, University of Manitoba, Islamabad, Pakistan; Health Services Academy	Institut de Recherche en Sciences de la Santé; Institut national de santé publique		Makerere University, School of Public Health	

* In Pakistan the term ‘therapeutic abortion’ was used to refer to legal, medically indicated abortion.

† In Burkina Faso the term ‘interruption sécurisée de grossesse autorisée par la loi’ or ‘safe termination of pregnancy authorized by law’ was used to refer to legally indicated abortion.

‡ Post-abortion care—including management of complications after an induced or spontaneous abortion—is legal in all countries.

HHFA, Harmonised Health Facility Assessment; SARA, Service Availability and Readiness Assessment.

To document lessons learnt, stakeholders met virtually to discuss challenges and successes from including abortion and/or post-abortion care in the SARA or HHFA. Stakeholders included individuals from WHO country offices, Ministries of Health, implementing partners, WHO regional offices and headquarters. These meetings took place in March to April 2023, after data collection and analysis were completed in all countries. When needed, an additional meeting addressed follow-up questions. In some meetings, all relevant stakeholders from a single country met simultaneously; in others, multiple meetings were held with individual stakeholders. The meetings were structured using a discussion guide and meetings were recorded. Two coauthors (HBJ and KF) attended each meeting and took detailed notes. These discussions are synthesised in the following sections, by stage of implementation.

### Deciding to integrate abortion into SARA and HHFA

The decision to integrate abortion into SARA and HHFA stemmed from a 4-year WHO multicountry project (2019–2022) focused on health system strengthening to prevent unsafe abortion and supporting quality abortion care.^22^ WHO country offices in Pakistan and Burkina Faso, engaged in the project, played a pivotal role in identifying opportunities to integrate comprehensive abortion care into national health information systems. Cross-country collaboration was fostered through the project, facilitating the Pakistan team to share insights with the Burkina Faso team in an August 2020 webinar, assuring the WHO country office and Ministry of Health in Burkina Faso of the feasibility of including an abortion section, even in contexts where abortion is stigmatised and legally restricted.

The timing coincided favourably with the development of the HHFA, and inputs from diverse health specialty areas were welcomed to address historically under-represented areas. Integrating different technical areas into global tools can be time-intensive and politically challenging for tool owners. It was therefore important to provide inputs during the global development stage, given the complexities of managing later tool updates. Following the inclusion of abortion and/or post-abortion care sections in HHFAs in Burkina Faso, Uganda and Zambia, abortion became part of the global HHFA core tool in 2022. Countries generally minimise adaptations, therefore we anticipate future HHFAs will continue to include abortion questions.

Despite our initial expectations of resistance at the country level due to concerns about the feasibility or abortion stigma, widespread support emerged for integrating abortion into SARA and HHFA across pilot countries. We attribute this to inclusive country-led survey coordination groups fostering pragmatic collaboration across different technical domains. Survey coordination groups, led by the Ministry of Health, provide leadership and oversight throughout the process, from defining survey objectives to determining inclusion and phasing of assessment questions, to ensuring dissemination and use of findings. Clear and consistent support from sexual and reproductive health leads along with thoughtful leadership from health information system leads in Ministries of Health and WHO offices facilitated integration of the abortion-related components.

### Questionnaire development and adaptations

The initial abortion section we used in the SARA in Pakistan was collaboratively designed by the WHO country and regional offices, supported by headquarters, using existing SARA service-specific sections, WHO abortion guidance^23^,^25^ and a review of post-abortion care assessment tools.^26^ Stakeholders from the Ministry of National Health Services Regulation and Coordination, provincial health departments and implementing partners reviewed and approved the final questions in a 2-day meeting covering the entire tool.^21^ A similar consultation process, led by Ministries of Health, was employed to adapt the abortion section for inclusion in the HHFA in each country. The indicator areas, as included in selected countries, are outlined in table 2. Questions were included in a specific abortion section, but also in relevant sections of the tool such as medicines and commodities, intimate partner violence services and charging and costs for services. The abortion section in each country was completed by questioning the ‘person most knowledgeable’ about the service, with questions asked in the area in the facility where abortion care was provided.

**Table 2:** Abortion and post-abortion care indicators included in the SARA and HHFA

	SARA	HHFA			
	Pakistan (2020–2021)	Burkina Faso (2020–2021)	Zambia (2020–2021)	Uganda (2022)	Ghana (2022–2023)
Abortion					
Availability of abortion care	Y	Y	Y		Y
Abortion care provided as inpatient/outpatient	Y	Y	Y		Y
Method of abortion care provided	Y	Y	Y		Y
Abortion care delivered in same area as deliveries			Y		
Whether/method of abortion care provided to adolescents	Y				Y
Number of abortion services provided in past year			Y		
Accuracy of registry for abortion procedures (facility-reported)			Y		
Existence of system to facilitate financial access (eg, sliding scale)			Y		
Whether fees are charged for abortion			Y		Y
Whether facility refers for abortion care		Y			
Support for abortion taking place in non-facility locations			Y		Y
Legal indications/conditions for which abortion is provided in facility		Y	Y	Y	Y
Abortion counselling offered to survivors of rape/sexual assault				Y	Y
Post-abortion care					
Availability of post-abortion care	Y	Y	Y	Y	Y
Post-abortion care provided as inpatient/outpatient	Y	Y	Y	Y	Y
Method of post-abortion care provided	Y	Y	Y	Y	Y
Post-abortion care delivered in same area as deliveries	Y	Y	Y		
Whether/method of post-abortion care provided to adolescents	Y	Y		Y	
Number of services provided for incomplete abortion in past year			Y		
Accuracy of registry for services provided for incomplete abortion (facility-reported)			Y		
Existence of system to facilitate financial access (eg, sliding scale)			Y		
Whether fees are charged for care for incomplete abortion			Y		Y
Whether facility refers for post-abortion care		Y			
Routine counselling for PAC and topics included in counselling		Y			
Post-abortion family planning					
Availability of post-abortion family planning	Y	Y	Y	Y	Y
Other ancillary services					
Counselling on sexually transmitted infections				Y	Y
Counselling on other health or support services, for example, gender-based violence or mental health				Y	Y
Service readiness					
Auditory and visual privacy for pre/post abortion care		Y	Y	Y	Y
Availability of national guidelines in the facility	Y	Y	Y	Y	Y
Register for recording services for abortion/post-abortion care		Y	Y	Y	Y
Recent training of providers of abortion/post-abortion care	Y		Y	Y	Y
Equipment for abortion/post-abortion care	Y	Y	Y	Y	Y
Medicines and products for abortion/post-abortion care	Y	Y	Y	Y	Y

Note: Ghana used the 2022 core tool, so this column indicates the contents of the core tool.

HHFA, Harmonised Health Facility Assessment; PAC, post-abortion care; SARA, Service Availability and Readiness Assessment.

In Pakistan, the SARA was implemented in two phases. In phase 1 we identified issues that informed subsequent data collection phases, including HHFA implementations in other countries. Initially, in Pakistan, we perceived resistance or discomfort among respondents regarding abortion questions. As abortion was the first section in the reproductive, maternal, newborn child and adolescent health (RMNCAH) part of the questionnaire, abortion availability was the first question asked to the designated RMNCAH respondent. In the second phase of data collection, we moved the abortion section further down in the RMNCAH section. Implementing teams reported that this facilitated rapport-building before sensitive questions were asked.

We found the terminology used to refer to abortion required careful attention. In Pakistan’s phase 1 we asked respondents if the facility offered ‘safe abortion (induced abortion) care’. In phase 2 we amended this to ‘safe abortion / therapeutic abortion / uterine evacuation care’, reassuring respondents that they were being asked about legal, medically indicated, services. Similarly, in Burkina Faso’s HHFA implementation, we referred to ‘safe termination of pregnancy authorized by law’. In Uganda, where abortion is legal in limited circumstances, the tool was adapted to include only questions about post-abortion care. Abortion services can be legally provided to save the life of the woman in Uganda, but this compromise was necessary for the tool to be perceived as locally acceptable while ensuring some relevant data (eg, on treatment of complications from unsafe abortion) were collected.

We found it was important to consider the level or type of facilities at which abortion questions would be asked. Initially, the Ministry of Health in Burkina Faso expressed hesitation to administer abortion questions in private facilities, given that abortion provision was authorised only in public sector teaching hospitals. However, policy revision was underway to strengthen the primary care provision of legal abortion and task sharing with nurses and midwives, in line with WHO guidelines.^3^ Under new national guidelines, private health facilities would be authorised to provide legal abortion. After consultation, the decision was made to administer abortion questions in all facilities, allowing HHFA data to serve as a baseline and be used to track changes following policy reform. In Zambia, skip patterns prevented abortion-related questions from being asked in lower-level facilities such as health posts. These skip patterns were standard practice in the Zambian HHFA for surgical interventions. However, abortion care does not necessarily require surgical intervention, and abortion care is legal in any health facility in Zambia if necessary to save the life or prevent injury to the physical or mental health of the pregnant person.^27^ This was resolved in the 2022 core tool update; now abortion questions are asked at all facility levels. These experiences highlight the need for consultation of and alignment with national policies and guidelines.

Finally, HHFA implementations in Burkina Faso, Uganda and Zambia revealed the tool’s overall length was unmanageable. The updated 2022 core tool was shortened, including the abortion section. For instance, detailed questions about both outpatient and inpatient provision of abortion were reduced to focus on outpatient care.

### Data collector training

Initially, we considered implementing a separate enumerator training on abortion to address issues of policy and stigma. Instead, we integrated abortion into RMNCAH training to avoid potential unintentional stigmatisation, or pragmatic exclusion for time-saving reasons. In some countries, we included abortion in training sessions on stigmatised areas of health like HIV, contraception and violence against women. We encouraged reflection on the rationale for including these topics in the survey. In some settings, we used role-play exercises to practice responding to participants who might be hostile or reluctant to report on stigmatised topics. We found that to be feasible, these exercises should be brief, include multiple stigmatised health topics and be facilitated by someone who is experienced, familiar with the local context and speaks the local language, for example, from local sexual and reproductive health civil society organisations.

Across all countries, we faced minimal issues with negative attitudes towards abortion among data collectors, likely due to the recruitment of experienced public health survey implementors, many of whom were medical professionals. Nevertheless, our experiences emphasised the importance of ensuring that supervisors and data collectors are well-informed that post-abortion care is always legal, about the indications for which abortion is legal and about which facilities should be prepared to provide these services. For example, in Ghana, data collectors were trained to probe further if respondents reported their facility did not offer any abortion-related care, as emergency treatment for complications from unsafe abortion cannot be denied. In Pakistan, where difficulties arose during abortion-related questioning in phase-1 data collection, phase-2 training incorporated more content on the legal status of abortion, on therapeutic, medically indicated, legal abortion and on post-abortion care. These adaptations were found to enhance enumerators’ confidence, and improve rapport with respondents.

### Implementation

During the process of implementation, we identified varied levels of sensitivity in responding to abortion-related questions and some strategies to manage discomfort. In Pakistan, data collectors noted unease among some respondents, leading enumerators to emphasise the assessment’s confidentiality. Female enumerators implemented the RMNCAH section in Pakistan, aiming to create a comfortable environment for the mostly-female gynaecologist respondents. In other countries, data collection raised fewer sensitivities, potentially related to differences in sociocultural norms or abortion’s legal context. For example, in Uganda only post-abortion care questions were included, a decision potentially contributing to fewer sensitivities. During pre-testing in Uganda, enumerators were attentive to respondent discomfort, yet reported no noticeable issues when posing post-abortion care questions, in both pre-testing and full implementation.

### Analysis

In the five countries, variations and some limitations exist in the domains and indicators assessing facility readiness for abortion or post-abortion care. Readiness is assessed for each facility by determining the number of criteria that are met across several domains, such as equipment, medicines, training and staffing. Notably, in Pakistan we included a ‘laboratory’ domain, although this is not necessary for routine abortion care.^3^ In Burkina Faso we excluded misoprostol from the readiness analysis, although this is an essential medication for abortion care. The 2022 HHFA core tool includes a standardised set of questions to assess abortion and post-abortion care service readiness, enhancing indicator consistency and facilitating cross-country comparisons. In future work, given the variety of methods used to calculate service readiness,^14 21 28^ an expert review process is needed to determine the most appropriate set of tracer indicators to include.

We identified issues with the aggregation of recommended and non-recommended methods. In Pakistan, dilatation and curettage (D&C) kits were grouped with vacuum aspirators. This was problematic at the analysis stage as D&C is not a WHO-recommended abortion method whereas vacuum aspiration is. Although corrected in HHFA questionnaires, the two methods were still grouped in reporting of service readiness in some countries. This underscores the need for sharing preliminary analyses and draft reports with experts to ensure alignment with global and national standards. However, this also partially reflects contextual differences in the health systems where the HHFA is conducted. For example, in Uganda, D&C and vacuum aspiration were not grouped together but were both included as indicators of service readiness, owing to very low levels of vacuum aspiration skills in the health workforce. Having D&C was considered better than having no method available; the report recommends transitioning from D&C to vacuum aspiration.^29^

### Dissemination and impact

The HHFA report should not sit in our drawers but should be used to further improve the performance of the health system in general and the provision of quality patient care in particular. (Burkina Faso HHFA report)^30^

Health facility assessment findings must be well-disseminated to inform policy and practice. In Pakistan, findings have informed district planning, the inclusion of misoprostol in routine district health information system (DHIS2) logistics monitoring and a commitment to increase health worker training in rural areas and urban informal settlements.^20^ This was partially achieved through opportunistic timing, as DHIS2 revisions were in progress when the SARA results became available. Delays in implementing these commitments due to funding challenges and the devastating floods of 2022, highlight difficulties in implementing evidence-based reforms.

To enhance impact, future HHFA technical and financial support could emphasise generating actionable plans from survey results. There is a need to go beyond the descriptive analysis offered by the HHFA tool, and ensure that Ministries of Health, subject-matter experts and local civil society are engaged in efforts to further explore the findings and their implications for programme improvement. Enhanced open access to SARA and HHFA data is needed to promote context-specific analysis and more informed decision-making. To this end, HHFA reports are increasingly available online, for example, in the Health Facility Assessment (HFA) data archive (the HFA data archive houses reports, data sets and supplemental material such as questionnaires, for countries that opt-in. See https://data-archive.hhfa.online/index.php/home).

### Summary

Lessons learnt from including abortion and/or post-abortion care in health facility assessments are summarised in box 1.

Box 1 **Key lessons learnt from incorporating the topic of abortion into health facility assessments**

**General principles contributing to the successful integration of the topic of abortion in the assessment:**

- Visible high-level political commitment from the beginning.
- Technical rigour.
- Robust questionnaire adaptation.
- Quality training.
- Quality data management.

**Specific factors contributing to the successful integration of the topic of abortion in the assessment:**

- Awareness and appreciation of roles of key stakeholders, including the leadership role of the Ministry of Health.
- Engagement of subject-matter experts throughout the assessment process promoted alignment of data, analyses and recommendations with national and global guidelines and relevance for the national context.
- Close working relationships among key stakeholders representing different technical areas contributed to:
  - Timely, relevant inputs into tool adaptations, including updating question sequencing and condensing of question set.
  - Use of nationally appropriate terminologies to refer to abortion.
  - Reference to national policies and guidelines in the assessment tool.
  - Integration of data collector training module on stigmatised health topics.
  - Alignment of analyses with national guidelines and policies, acknowledgement of deviations from global guidelines.
  - Cross-country learning and experience sharing.

## Conclusion

Our experience including an abortion section in the SARA and HHFA demonstrates feasibility across a range of countries and abortion legal contexts. We experienced less resistance than expected in implementing the abortion questions, which suggests those working in neglected health areas should identify opportunities to include topics in monitoring tools, particularly when tool adaptations are being planned or underway.

To some extent, the principles for including abortion in health facility assessments are common across specific health topic areas: it is essential to have a visible high-level commitment from the beginning, technical rigour, robust questionnaire adaptation, quality training and quality data management.

For abortion to be included successfully, we have identified specific facilitating factors, including appreciation of roles of key stakeholders (leadership or advisory), close working relationships among key stakeholders, cross-country learning, timely input into tool adaptations, use of locally appropriate terminology to refer to abortion and reference to national policies and guidelines. In the design stage, it is important to consider question sequencing and to avoid excessive lengthening of the overall tool. In data collector training, we found it is key to balance normalisation of abortion with the need to sensitise and educate prior to data collection so that enumerators are equipped to manage respondent reluctance to answer questions on stigmatised health areas and are aware of the legal indications for abortion. Our work highlights the need to ensure adequate disaggregation of recommended and non-recommended abortion methods, to align analysis with national guidelines and policies, to develop standardised measures for service readiness and to use findings to generate evidence-based action plans. Finally, engagement of subject-matter experts throughout the process is key to ensuring data and analyses and recommendations are aligned with global and national guidelines, and relevant to the national context.

Abortion is an essential health service that should be included in core health facility assessment tools and national facility surveys. Including abortion in WHO health facility assessment tools has provided important data to inform policy and programmes, can help to normalise abortion as healthcare and clarifies the conditions under which this service can be provided.

## Data Availability

Data are available upon reasonable request.
